# Effect of home-based cardiac rehabilitation on quality of life, health behaviours and cardiac anxiety in patients with coronary artery disease: findings from a single-blinded randomised controlled trial

**DOI:** 10.1136/bmjopen-2025-114349

**Published:** 2026-06-30

**Authors:** Adnan Yaqoob, Laila Ladak, Aamir Hameed Khan, Aysha Almas, Asif Hanif, Furqan Yaqub Pannu, Faizan Khemani, Wajeeha Sahar, Rubina Barolia

**Affiliations:** 1 School of Nursing and Midwifery, The Aga Khan University, Karachi, Sindh, Pakistan; 2 Department of Nursing Education, Shaukat Khanum Memorial Cancer Hospital and Research Centre, Lahore, Punjab, Pakistan; 3 Lahore School of Nursing, The University of Lahore, Lahore, Punjab, Pakistan; 4 Department of Cardiology, Ziauddin University, Karachi, Sindh, Pakistan; 5 Department of Medicine, The Aga Khan University, Karachi, Sindh, Pakistan; 6 University Institute of Public Health, The University of Lahore, Lahore, Punjab, Pakistan; 7 Department of Cardiology, Punjab Institute of Cardiology, Lahore, Punjab, Pakistan; 8 Faculty of Health Sciences, The Aga Khan University Medical College Pakistan, Karachi, Sindh, Pakistan; 9 Department of Rehabilitation Sciences, The University of Faisalabad, Faisalabad, Punjab, Pakistan

**Keywords:** CARDIOLOGY, Myocardial infarction, Rehabilitation medicine, Quality of Life, Behavior, Cardiovascular Disease

## Abstract

**Objective:**

To evaluate the effectiveness of a contextually developed home-based cardiac rehabilitation (HBCR) programme on heart-related quality of life (QoL), cardiac health behaviours (CHB) and cardiac anxiety (CA) among patients with coronary artery disease (CAD) in Lahore, Pakistan.

**Design:**

Single-blinded randomised controlled trial (RCT).

**Setting:**

Cardiology department of a public tertiary-care hospital in Lahore, Pakistan.

**Participants:**

120 patients aged 18–65 years diagnosed with coronary artery disease who had undergone percutaneous coronary intervention or medical management were recruited and randomly allocated to intervention (n=60) and control (n=60) groups.

**Intervention:**

Participants in the intervention group received a nurse-led HBCR programme consisting of discharge education, structured physical activity and exercise guidance, dietary counselling, medication adherence support, and regular telephonic and physical follow-ups over 24 weeks. The control group received routine care and standard discharge advice.

**Primary outcome measures:**

Primary outcomes were heart-related quality of life (MacNew HRQoL), cardiac health behaviours (Cardiac Health Behaviour Scale-21) and cardiac anxiety (Cardiac Anxiety Questionnaire-18), assessed at baseline, 3 months and 6 months postdischarge.

**Results:**

At 6-month follow-up, the intervention group demonstrated significantly higher global QoL scores compared with the control group (mean difference 30.71, 95% CI 22.90 to 38.50). CHB scores were also significantly higher in the intervention group (mean difference 19.60, 95% CI 16.20 to 23.00). CA scores were significantly lower among participants receiving HBCR (mean difference −18.72, 95% CI −21.00 to −16.40). These improvements were evident after 3 months and sustained at 6 months.

**Conclusion:**

The nurse-led HBCR programme significantly improved QoL and CHB and reduced CA among patients with CAD. HBCR may provide an effective and scalable secondary prevention strategy in settings where centre-based cardiac rehabilitation services are limited.

**Trial registration:**

Australian New Zealand Clinical Trial Registry, ACTRN12623000049673p.

**STRENGTHS AND LIMITATIONS OF THIS STUDY**

- A single-blind randomised controlled trial design was employed to reduce selection bias.
- The home-based cardiac rehabilitation intervention was tailored to the local sociocultural context, which makes it more acceptable to patients with CAD.
- Valid and reliable tools were used to collect data on outcomes, that is, QoL and Cardiac Health Behaviour Scale-21, from participants.
- Outcomes were based on self-reported measures, which may introduce bias.
- The follow-up duration of 6 months may not fully capture the long-term sustainability of behavioural changes or their potential impact on recurrent cardiovascular events.

## Introduction

 Home-based cardiac rehabilitation (HBCR) is an alternative model of cardiac rehabilitation (CR) in which structured rehabilitation activities are delivered in the patient’s home environment rather than in hospital-based rehabilitation centres. HBCR programmes typically include components such as physical activity (PA) and exercise guidance, dietary counselling, medication adherence support, psychological support, and regular monitoring through telephonic or remote follow-up. However, evidence from low-income and middle-income countries (LMICs) remains limited, particularly in contexts where access to centre-based cardiac rehabilitation (CBCR) is restricted.

The existing CBCR services have several barriers that are common among LMICs, such as financial constraints, transportation issues, work schedule conflicts and low health literacy levels, which prevent many patients from participating in CBCR programmes.^1 2^ In a scoping review conducted by Iyngkaran *et al*, data from 31 297 patients with acute myocardial infarction (AMI) showed that the distance to a CR facility was the most common barrier (OR 1.75, 95% CI 1.64 to 1.86); patients who lived >16 km away from CR service had 25% higher CR non-attendance, whereas those with >50 km of distance from a cardiac rehabilitation facility had threefold higher chances of non-attendance. Transportation and distance to CR centres were also the most perceived barriers among patients (68%) when explored qualitatively.^3^

Globally, 55% of countries offer CR programmes. There is only one CR spot available for 12 patients with CAD in high-income countries, as compared with 66 patients who require CR in LMICs. Moreover, the existing CR programmes in Pakistan have a capacity of 6000 spots per year, with 622 146 schemic heart disease (IHD) incidents, making an annual programme capacity of one CR spot for 104 patients,^4^ whereas Pakistan needs 616 146 more spots per year to meet the CR needs.^5^ This poses a risk of secondary cardiac events, which ultimately increases the burden on tertiary care hospitals.

CR programmes are designed not only to reduce cardiovascular risk factors and prevent recurrent cardiac events but also to improve patients’ functional capacity, psychological well-being and overall health-related QoL (HRQoL).^6^ Patients recovering from MI often experience reduced physical functioning, anxiety and lifestyle limitations that can negatively affect QoL. CR interventions, including home-based models, have been shown to improve QoL through structured PA, lifestyle counselling and psychosocial support.^7^ Therefore, assessing changes in QoL provides an important patient-centred measure of the effectiveness of CR interventions.

Therefore, this study aimed to evaluate the effectiveness of a nurse-led HBCR programme among patients with CADs in Lahore, Pakistan. The primary objectives were to assess the effect of HBCR on HRQoL, CHB and CA at 6 months following discharge.

## Methods

### Design

This study was a single-blinded parallel group randomised controlled trial. The trial was prospectively registered with the Australian New Zealand Clinical Trials Registry (ANZCTR; ACTRN12623000049673p). The detailed study protocol, including the intervention development process and trial procedures, has been published previously.^8^ There were no substantive deviations from the published protocol. While all prespecified primary outcomes are reported in this manuscript, prespecified secondary outcomes will be reported separately. This manuscript was prepared and reported in accordance with the consolidated standards of reporting trials (CONSORT) 2025 guidelines.^9^

### Trial setting

The participants were recruited from the inpatient cardiology departments of Mayo Hospital, Lahore, Pakistan, between February and May 2024. Mayo Hospital is a public sector tertiary care hospital. It has a capacity of 2399 beds, with 52 beds specified for cardiac patients. Each participant was followed for 24 weeks (6 months) after discharge, with outcome assessments conducted at baseline, 3 months and 6 months. The assessment of the last participant was completed in October 2024.

### Eligibility criteria

#### Inclusion criteria

- Patients between 18 years and 65 years of age
- Male and female
- Patients diagnosed with acute ST-segment Elevated Myocardial Infarction (STEMI), Non ST-segment Elevated Myocardial Infarction (NSTEMI) and/or angina
- Patients underwent first percutaneous coronary intervention (PCI) and/or were managed medically
- Patients with New York Heart Association classification for dyspnoea I and II
- Patients residing within 50 km of the Mayo Hospital, Lahore
- Patients who can understand, read and speak Urdu.

#### Exclusion criteria

- Patients with left ventricular ejection fraction <50%
- Patients with resting systolic Blood Pressure (BP) >200 mm Hg or resting diastolic BP >110 mm Hg
- Patients with signs and symptoms of postprocedure ischaemia
- Patients who have been prescribed restricted mobility due to comorbidities such as aortic stenosis and dysrhythmias
- Patients who have altered QoL due to comorbidities, such as end-stage renal disease, liver failure and respiratory failure.

### Control group (routine care)

**Figure 1:**
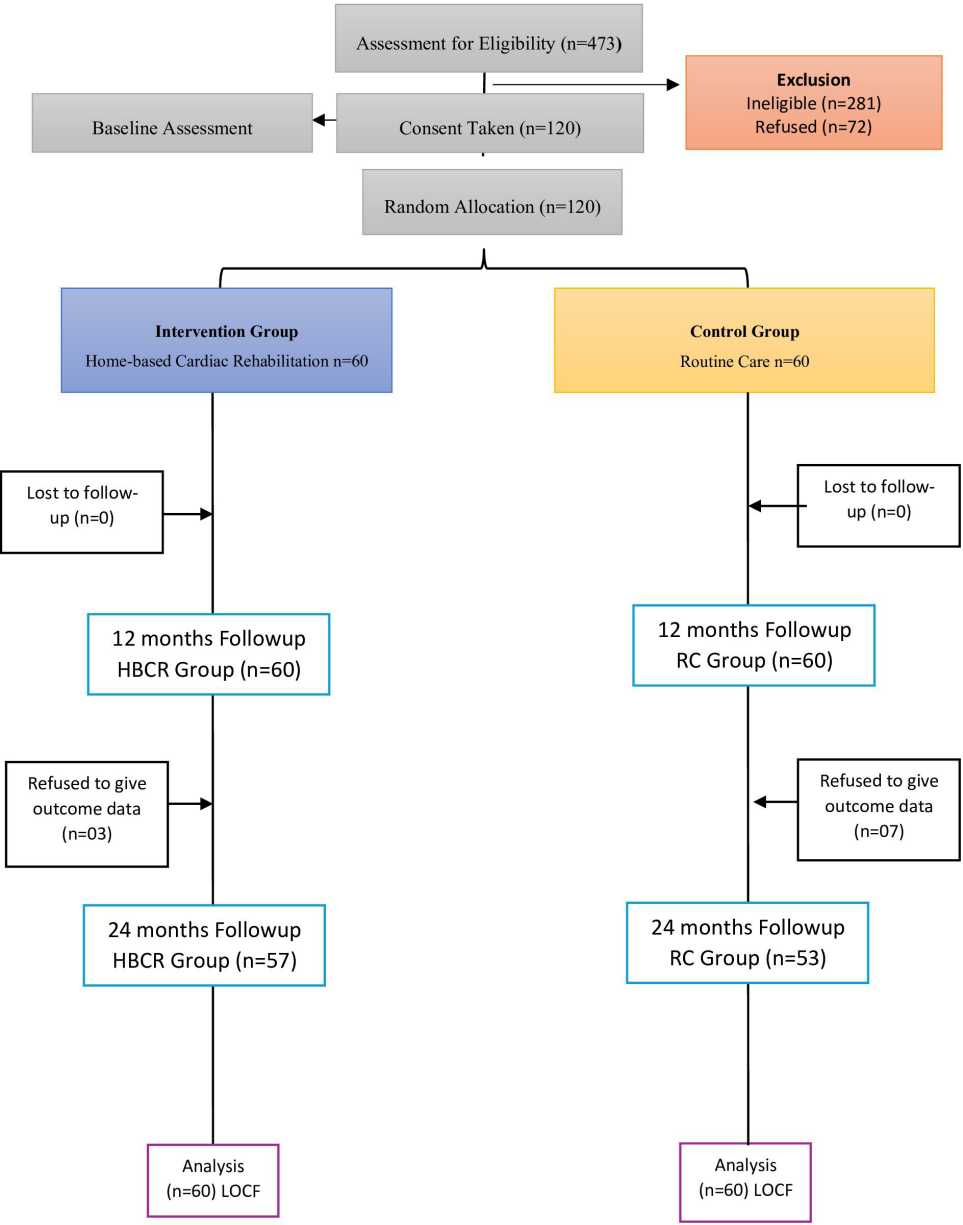
CONSORT (Consolidated Standards of Reporting Trials)diagram. HBCR, home-based cardiac rehabilitation; LOCF, Last Observation Carried Forward.

Participants in the control group received the usual care. The usual care for this study was defined as ‘following the cardiac procedure, patients are discharged and called for first follow-up after 14 days with no structured plan of subsequent follow-ups’. Participants in the control group were also provided with the HBCR module hard copy only, a calendar mentioning the follow-up dates (ie, on 14 days, week 12 and week 24), and routine teaching on their discharge. There was no cardiac rehabilitation facility available in the study setting. The routine care was administered by face-to-face consultation, physically on their scheduled follow-ups, by the routine cardiologists; however, the researcher followed them on their visits to collect the postassessment data for up to 6 months (at the third and sixth months). Participants in the control group were called for a reminder of the follow-up a day before to obtain postassessment data. All the participants provided data at first follow-up (figure 1). They were also provided with financial compensation for the scheduled physical follow-up in the hospital.

### HBCR intervention

Participants allocated to the intervention group received an HBCR programme delivered over 24 weeks following hospital discharge. The programme focused on the management of cardiovascular disease (CVD) risk factors, including healthy diet, physical activity, exercise, medication adherence, weight management, smoking cessation and anxiety management. Discharge education was initially provided by a trained cardiac nurse, and participants were given an HBCR booklet containing educational material and guidance for lifestyle modification and recovery. The intervention booklet and programme structure were developed based on the expert opinion of a multidisciplinary team including cardiologists, nurses, nutritionists, physiotherapists, psychologists and pulmonologists.

The HBCR programme consisted of three phases. *Phase I* began during hospitalisation, during which risk factors were identified, and discharge counselling was provided by a nurse. Participants received the HBCR booklet and a follow-up calendar outlining scheduled contacts. The first physical follow-up visit was scheduled 1 week after discharge.

*Phase II* began at the first postdischarge physical follow-up and continued from week 1 to week 12. These visits were conducted in the presence of a physician, led by a nurse researcher, and a trained nurse. During this phase, three physical follow-up visits and six telephonic follow-ups were scheduled. At the first physical follow-up, participants received training on physical activity and exercise. Patients were provided with a pulse oximeter and were trained in its proper use to monitor heart rate and achieve the target heart rate during walking exercise. Participants demonstrated correct device usage using the teach-back method. A personalised physical activity and exercise plan was provided for 1 month. Reinforcement of intervention components was provided through telephone calls, text messages and WhatsApp reminders in Urdu, delivered to participants’ mobile phones.

*Phase III* extended from week 13 to week 24. During this phase, participants received three physical follow-up visits and three telephonic follow-ups. Patients were encouraged to continue adherence to recommendations related to PA and exercise, dietary modifications, weight management, anxiety management, smoking and tobacco cessation, and medication adherence.^9^

The HBCR intervention was delivered primarily by the researcher, who coordinated the programme and conducted the educational counselling and follow-up sessions. The cardiac nurse also assisted with telephonic follow-ups and reminder calls to reinforce adherence to the intervention components and scheduled visits. Physical follow-up visits were conducted in the presence of a physician, who provided clinical oversight when required. Intervention fidelity was maintained through the use of a structured follow-up calendar, scheduled physical and telephonic contacts, reminder messages, and documentation of participant engagement throughout the intervention period. The detailed schedule and components of the HBCR intervention are presented in table 1.

**Table 1:** HBCR intervention

HBCR	Phase I	Phase II												Phase III											
		Weeks																							
	During hospitalisation	1	2	3	4	5	6	7	8	9	10	11	12	13	14	15	16	17	18	19	20	21	22	23	24
Recruitment	**✓**																								
Counselling about risk factors, diet, weight, anxiety, medication management, PA, smoking and psychological support	**✓**	**✓**				**✓**				**✓**				**✓**				**✓**				**✓**			
HBCR booklet	**✓**																								
Physical follow-up		**✓**				**✓**				**✓**				**✓**				**✓**				**✓**			
Training for PA and exercises		**✓**																							
THR goal setting		**✓**				**✓**				**✓**				**✓**				**✓**				**✓**			
Text messages for reminder				**✓**				**✓**				**✓**				**✓**				**✓**					
Text messages containing intervention details			**✓**	**✓**	**✓**		**✓**	**✓**	**✓**		**✓**	**✓**	**✓**		**✓**	**✓**	**✓**		**✓**	**✓**	**✓**		**✓**	**✓**	**✓**
Telephonic follow-ups			**✓**	**✓**	**✓**		**✓**		**✓**				**✓**				**✓**				**✓**				**✓**
Outcome data	**✓**													**✓**											**✓**

HBCR, home-based cardiac rehabilitation; PA, Physical Activity; THR, Target Heart Rate.

### Outcomes

The primary outcomes were heart-related QoL, cardiac health behaviours and cardiac anxiety, assessed using the MacNew Heart Disease Health-Related Quality of Life Questionnaire, the Cardiac Health Behaviour Scale (CHB-21) and the Cardiac Anxiety Questionnaire (CAQ-18), respectively. Outcomes were measured at baseline, 3 months and 6 months after discharge. The 6-month follow-up was considered the primary endpoint, while the 3-month assessment was used to examine the interim effects of the intervention. Secondary outcomes from the study will be reported elsewhere.

#### Quality of life

QoL was measured through a licensed Macnew health-related QoL instrument (Urdu version). It is a reliable tool for assessing the QoL in patients post MI with Cronbach alpha coefficients of 0.95, 0.93 and 0.95 in emotional, physical and social domains, respectively.^10^ The instrument consists of 27 items with a 7-point Likert Scale. The minimum score is 27, while the maximum is 189. The tool’s reliability was also checked on the Pakistani population (n=20), which was 0.89. The QoL was measured at baseline, 90 days and 180 days.

#### Cardiac health behaviours

The health behaviours of cardiac patients were measured by the CHB-21. It has 21-items covering five dimensions: Health Responsibility, PA, Stress Management and Smoking Cessation (SmC), with a 5-point Likert Scale. The higher scores mean highly protective behaviour. The tool is reliable, with a Cronbach’s alpha of 0.82.^11^ However, when it was converted to Urdu by field experts, it was checked for validity and reliability. For Content Validity Index (CVI), the scale was distributed to five experts, including a cardiologist, a nurse, a physiotherapist, a nutritionist and a psychologist with more than 5 years of experience. The CVI of the tool was 0.92, whereas, for reliability, the tool was administered to 20 patients with CVD. The Cronbach’s alpha of the tool was 0.82. The CHB was measured at baseline, 90 days and 180 days. Based on the literature, the following cut-off was used to report CHB categories.

#### Cardiac anxiety

Anxiety related to cardiac events/procedures was checked through a validated questionnaire, that is, CAQ-18. This scale has 18 items with a 5-point Likert Scale starting from 0=Never to 4=Always. The minimum score is 0, while the maximum is 72. The higher scores indicate severe CA. The tool is reliable, with a Cronbach’s alpha of 0.86.^12^

The tool was converted into Urdu by experts and checked for validity. For CVI, it was distributed to five HCPs, including two clinical psychologists, a cardiologist, a nurse and a physiotherapist. The CVI of the tool was 0.96. For reliability, the tool was administered to 20 patients with CAD, and the alpha coefficient was calculated to be 0.90. The CA was measured at baseline, 90 days and 180 days.

For analysis, responses to each item were scored according to the original scale instructions, and total scores were calculated by summing individual item scores, with higher scores indicating better cardiac health behaviours (CHB-21) and greater cardiac anxiety (CAQ-18), respectively.

### Harms

As the intervention involved a structured home-based rehabilitation programme consisting primarily of education, lifestyle counselling and guided physical activity, no major intervention-related harms were anticipated. Participants were instructed to report any adverse symptoms or health concerns during follow-up visits or telephonic contacts with the research team. Monitoring was done for any adverse events during the intervention period, including symptoms such as chest pain, injury during exercise or any medical events requiring hospital admission.

### Sample size

Sample size calculations were initially performed for all three prespecified primary outcomes of the study. A formal multiplicity adjustment was not applied; instead, the study was conservatively powered based on the primary outcome requiring the largest sample size. Among the primary outcomes, heart-related QoL required the largest sample size and was therefore used to determine the final sample size for the trial. Estimates for the expected mean difference and SD were derived from a previously published study of patients with coronary heart disease that assessed QoL,^13^ which reported mean scores of 80.8 (SD±13.7)^13^ in the intervention group and 73.2 (SD±13.0)^13^ in the control group at 6 months. Assuming a two-sided significance level of 0.05 and 80% statistical power, the required sample size was calculated as 49 participants per group. To account for an anticipated 20% loss to follow-up, the calculated minimum sample size was 59 participants. To facilitate equal allocation, the sample size was rounded up to 60 participants per group, resulting in a total sample size of 120 participants.

### Randomisation

Participants who met the eligibility criteria and provided informed consent were randomly allocated to the intervention or control group using an envelope-based allocation procedure. Before recruitment, group allocation codes representing the intervention and control groups were prepared and placed inside opaque sealed envelopes. The envelopes were mixed thoroughly and kept by an independent allocator who was not involved in participant recruitment or outcome assessment. After baseline data collection, the allocator randomly selected and opened one envelope for each participant and assigned the corresponding study code. The researcher maintained a confidential list linking participant identifiers with their assigned codes. This procedure ensured allocation concealment during the recruitment process. To smoothen the random allocation process, the researcher worked closely with the cardiology team to develop a referral plan for cardiac rehabilitation evaluation.

### Blinding

The independent outcome assessors were blinded to group assignment. In health, behaviour and rehabilitation interventions, blinding the investigator/researcher or participants is difficult; the outcome assessor can be blinded to the group assignment.^14^ The outcome assessors were hired and trained by the research team. They were trained to collect the baseline data for QoL, health behaviours, biophysiological parameters and CA through validated questionnaires soon after the informed consent was obtained and before the allocation took place. Different assessors were hired for each time point, that is, baseline, third month and sixth month. The collected data were segregated with the help of secret coding. A room was designated for the researcher by the hospital administration, where the follow-up meetings were conducted with patients. The intervention lasted up to 24 weeks after discharge. An interventional cardiologist facilitated and supervised the physical follow-up during the entire intervention. Participants’ eligibility, selection, retention and dropouts are presented in the CONOSRT flow diagram (figure 1).

### Data entry and analysis

Data were entered and analysed using IBM SPSS V.24. Descriptive statistics were used to summarise participant characteristics and outcome variables. Continuous variables were presented as means and SD, while categorical variables were presented as frequencies and percentages.

The distribution of continuous outcome variables was assessed using the Kolmogorov–Smirnov test and visual inspection of histograms (online supplemental file 1). The MacNew HRQoL, CHB-21 and CAQ-18 instruments generate composite scores by summing responses across multiple Likert-type items. When Likert-type items are aggregated into composite scale scores, the resulting variables are commonly treated as approximately continuous and analysed using parametric statistical methods in health research.^15^

Baseline characteristics between the intervention and control groups were compared using an independent sample t-test and χ^2^ tests. Independence of observations was ensured through the randomised study design. The assumption of sphericity for repeated-measures analysis of variance (ANOVA) was assessed using Mauchly’s test, which indicated that the assumption was not violated (χ² = 4.639, df=2, p=0.098). Therefore, the standard repeated-measures ANOVA results were used to evaluate differences between groups over time. This approach allowed assessment of within-group changes over time and between-group differences across the three measurement points (baseline, 3 months and 6 months). The results are reported as between-group mean differences with corresponding 95% CIs, and statistical significance was set at p<0.05.

#### Internal validity

Several strategies were implemented to minimise threats to internal validity. Eligible participants were randomly allocated to the intervention and control groups to overcome selection bias. The HBCR intervention was delivered using a structured Urdu-language booklet and telephonic follow-up protocols to ensure consistency across participants. Validated instruments were used with excellent reliability. Baseline and follow-up assessments were conducted at predetermined intervals (0, 90 and 180 days). While blinding of participants was not feasible due to the behavioural nature of the intervention, the data assessors were blinded to group allocation during analysis to mitigate detection bias. Response rates were high in both groups (95% intervention, 88% control), which helped reduce the risk of attrition bias. Missing data were minimal and managed appropriately.

### External validity

Although the study was conducted at a single tertiary care hospital in Lahore, several measures were taken to enhance the transferability of findings. The intervention was co-developed with patients and healthcare professionals to ensure cultural relevance. Recognising family roles in Pakistani society, caregivers were engaged to support adherence and informational validity. The use of Urdu, the national language, increases the potential adaptability of the materials in other regions of Pakistan. A rich description of the setting, participant characteristics and intervention processes was provided to allow assessment of applicability to other similar contexts.

### Patient and public involvement

Patients and healthcare professionals were involved in the development of the HBCR intervention through qualitative interviews conducted during the initial phase of the study. Insights from patients and healthcare providers helped inform the content and structure of the HBCR programme to ensure that it was contextually appropriate and feasible in the local setting. Patients were not involved in the recruitment process, data collection or outcome assessment during the randomised controlled trial phase. The findings of the study have been disseminated through presentations at research symposia and will also be shared with participating healthcare professionals and study participants through summary reports at the study site.

## Results

A total of 120 participants were randomised, with 60 allocated to the HBCR group and 60 to the control group. All participants in the intervention group received the intended intervention at the third month. Outcome data for the primary outcomes were available for 57 participants in the intervention group and 53 participants in the control group at 6 months, and these participants were included in the final analysis. Their analysis was done through the Last Observation Carried Forward method. The participants who refused to provide data at the sixth month showed the reason of ‘no longer interested’.

Table 2 compares the baseline characteristics of participants in the control and intervention groups. The mean age of participants in the control group was 52.30 (SD±9.60) years, whereas in the intervention group, it was 50.20 (SD±9.10) years. There were 76.7% (n=46) male and 23.3% (n=14) female participants in the control group, while 70% (n=42) were male and 30% (n=18) were female in the intervention group. Data regarding the diagnosis revealed that the majority of participants in the control group, 53.3% (n=32), were diagnosed with STEMI, 26.7% (n=16) were diagnosed with angina and 20% (n=12) were diagnosed with NSTEMI. In the intervention group, similarly to the control group, the majority of participants, 58.3% (n=35), were diagnosed with STEMI, followed by NSTEMI (26.7% n=16) and angina (15% n=9). Furthermore, regarding hypertension, 60% (n=36) were known cases in the control group and 48.3% (n=29) in the intervention group. Moreover, regarding diabetes mellitus, 35% (n=21) and 41.7% (n=25) were diabetic in the control and intervention groups, respectively.

**Table 2:** Baseline characteristics

Variables		Control group	Intervention group
Age		M 52.30, SD±9.60	M 50.20, SD±9.10
Sex	Male	46 (76.7%)	42 (70%)
	Female	14 (23.3%)	18 (30%)
Diagnosis	Angina	16 (26.7%)	09 (15%)
	NSTEMI	12 (20.0%)	16 (26.7%)
	STEMI	32 (53.3%)	35 (58.3%)
HTN	No	24 (40%)	31 (51.7%)
	Yes	36 (60%)	29 (48.3%)
DM (type II)	No	39 (65%)	35 (58.3%)
	Yes	21 (35%)	25 (41.7%)
Weight		M 73.95, SD±11.05	M 75.07, SD±11.16
QoL		M 106.70, SD±19.96	M 103.08, SD±21.47
CHB		M 39.48, SD±11.37	M 41.06, SD±9.76
CA		M 30.21, SD±13.62	M 31.48, SD±15.28
Total cholesterol		M 192.96, SD±49.69	M 202.94, SD±58.82

CA, Cardiac Anxiety; CHB, Cardiac Health Behaviour Scale; DM, Diabetes Mellitus; HTN, Hypertension; NSTEMI, Non ST-segment Elevation Myocardial Infarction; QoL, quality of life; STEMI, ST-segment Elevation Myocardial Infarction.

Table 3 presents the comparison of global QoL, cardiac health behaviours (CHBs), and cardiac anxiety between the control and intervention groups at baseline, 90 days and 180 days. At baseline, there were no meaningful differences between the groups in any of the measured outcomes. The mean global QoL Score was 106.70±19.96 in the control group and 103.08±21.47 in the intervention group, with a mean difference of −3.62 (95% CI −11.10 to 3.90). Similarly, CHB scores were comparable between groups (mean difference 1.58; 95% CI −2.10 to 5.30), as were cardiac anxiety scores (mean difference 1.27; 95% CI −3.90 to 6.40), indicating baseline equivalence.

**Table 3:** Effects of HBCR on QoL and CHB

Outcome variables	Frequency of observations	Control group n=60	Intervention group n=60	*Mean difference*	*95%* CI	*P Value*
QoL global	Baseline	106.70±19.96	103.08±21.47	−3.62	−11.10 to 3.90	<0.001
	90 days	128.46±19.09	165.15±14.16	36.69	30.60 to 42.80	
	180 days	141.95+25.18	172.66±15.08	30.71	22.90 to 38.50	
**CHB**	Baseline	39.48±11.37	41.06±9.76	1.58	−2.10 to 5.30	<0.001
	90 days	42.70±10.76	62.65±8.31	19.95	16.50 to 23.40	
	180 days	45.53±9.87	65.13±8.27	19.60	16.20 to 23.00	
Cardiac anxiety	Baseline	30.21±13.62	31.48±15.28	1.27	−3.90 to 6.40	<0.001
	90 days	35.58±10.20	21.18±10.93	−14.40	−18.20 to −10.60	
	180 days	30.35±6.24	11.63±6.75	−18.72	−21.00 to −16.40	

Between-group differences were analysed using repeated-measures analysis of variance (ANOVA). Statistical significance was set at p<0.05 for the group × time interaction of each outcome.

CHB, Cardiac Health Behaviour Scale; HBCR, home-based cardiac rehabilitation; QoL, quality of life.

At 90 days, the intervention group demonstrated significantly greater improvements compared with the control group. Global QoL scores were substantially higher in the intervention group (165.15±14.16) compared with the control group (128.46±19.09), corresponding to a mean difference of 36.69 (95% CI 30.60 to 42.80). Cardiac health behaviour scores were also significantly higher in the intervention group (62.65±8.31) than in the control group (42.70±10.76), with a mean difference of 19.95 (95% CI 16.50 to 23.40). In contrast, cardiac anxiety scores were significantly lower in the intervention group (21.18±10.93) compared with the control group (35.58±10.20), with a mean difference of −14.40 (95% CI −18.20 to −10.60).

These differences persisted at 180 days. The intervention group continued to demonstrate higher global QoL scores (172.66±15.08) compared with the control group (141.95±25.18), with a mean difference of 30.71 (95% CI 22.90 to 38.50). Similarly, CHB scores remained higher in the intervention group (65.13±8.27) than in the control group (45.53±9.87), corresponding to a mean difference of 19.60 (95% CI 16.20 to 23.00). Cardiac anxiety remained significantly lower in the intervention group (11.63±6.75) compared with the control group (30.35±6.24), with a mean difference of −18.72 (95% CI −21.00 to −16.40). Repeated-measures ANOVA showed a significant group × time interaction for QoL, CHB and CA (p<0.001), indicating that improvement over time, that is, a 6-month follow-up period, was significantly greater in the HBCR group compared with the control group.

Overall, the intervention group showed substantial and sustained improvements in QoL and cardiac health behaviours, along with significant reductions in cardiac anxiety, compared with the control group over the 6-month follow-up period.

With respect to adverse events, no physical harm, such as chest pain and injury, was reported by participants related to the intervention group, which could require emergency admission. However, at 6 months, in the intervention group, 2 participants had emergency visits as compared with 10 in the control group due to non-cardiac reasons. Reduction in emergency visits in the intervention group in the current study is attributed to improved patient interaction with the researcher and healthcare professionals (HCPs), telephonically addressed health concerns with the help of a cardiologist, physical health, weight loss and better control of cardiac risk factors, all of which are optimised through HBCR with enhanced participation over 6 months.

## Discussion

The findings showed that the patients who received HBCR substantially improved their QoL. Additionally, the notable improvements in QoL in the intervention group are in line with the literature. For instance, Zheng *et al* reported that structured HBCR can significantly improve the QoL of patients with CAD.^7^ Similarly, it is also consistent with a recent study that demonstrated the positive effect of tailored interventions on the QoL of cardiac patients.^16^ The improvement in Global-QoL (baseline; 103.08+21.47 vs 180 days: 172.66+15.08) in the intervention group can be attributed to PA and exercise training, monitoring, and encouragement, dietary instructions, timely counselling, improved communication and psychological support. However, improvement in the control group may be attributed to the provision of a booklet, which might have benefited them. Further, the improvement in QoL in the current study is also in line with the research that indicates cardiac rehabilitation participation is a critical element in the recovery process and overall health of cardiac patients.^6^ Additionally, a meta-analysis conducted by Dibben *et al* in 2023 included 84 RCTs covering 23 430 participants with CVD and found that there was a significant improvement in HRQoL and risk factor reduction among patients who participated in cardiac rehabilitation.^17^

The improvements observed in heart-related QoL were not only statistically significant but also clinically meaningful. Previous research using the MacNew HRQoL instrument suggests that a change of approximately 0.5 points per item represents the minimal clinically important difference (MCID).^18^ In the present study, the intervention group demonstrated an improvement of approximately 2.6 points per item, whereas the control group improved by approximately 1.3 points per item between baseline and 6 months. These improvements substantially exceeded the established MCID threshold, indicating that participation in the HBCR programme produced clinically meaningful benefits in patients’ perceived health status.

Similarly, the rise in CHB in the intervention group was significant and supports the findings of Chen *et al*, whose research established that patients who underwent HBCR exhibited higher levels of adherence to recommended health behaviours, including exercise and dietary modification.^19^ However, due to minimal behaviour change recommendations in Pakistan, patients often feel as if no change is required; further, they continue with the previous behaviours with the inclusion of certain medicines in their routine.^20^

Improvements were also observed in cardiac health behaviours. Since MCID has not been formally established for the CHB-21 and CAQ scales, the magnitude of change was interpreted using a distribution-based approach. Methodological studies have shown that approximately 0.5 SD represents a reasonable estimate of a clinically meaningful change in health-related outcomes.^21^ Based on the baseline SD of CHB scores in the present study (SD=9.76), the estimated minimally important difference was approximately 4.9 points. The intervention group demonstrated an improvement of 24.07 points between baseline and 6 months (41.06 to 65.13), which substantially exceeded this estimated threshold, suggesting that the HBCR intervention produced a clinically meaningful improvement in cardiac health behaviours.

Regarding CA, the intervention group exhibited a significant reduction in anxiety scores, with significant differences at 90 days (p<0.001) and 180 days (p<0.001). The reduction is consistent with the findings of recent studies, which also suggest that cardiac rehabilitation programmes are responsible for the reduction in anxiety among patients with CAD.^22^ Additionally, similar insights are found in the literature. A study from Australia reported that 15% of the participants who had moderate anxiety levels before the cardiac rehabilitation intervention improved to normal anxiety at the end of cardiac rehabilitation.^23^ On the contrary, findings reported by Olsen *et al* from Norway revealed no difference in anxiety levels between cardiac rehabilitation and non-cardiac rehabilitation groups over 3 years, with a 32% prevalence of anxiety in 775 patients who underwent PCI for the first time.^24^

The reduction in anxiety in the intervention group can be attributed to the holistic support provided throughout HBCR, which combines physical exercise, encouragement, psychological support and anxiety-relieving techniques with frequent counselling sessions. The findings of the study suggest that HBCR is responsible for the reduction in the emotional burden of CAD, a finding that is of special significance in the Pakistani context, where psychological distress related to chronic illness is often not well addressed. Using the baseline SD of cardiac anxiety scores in the present study (SD=15.28), the estimated minimally important difference was approximately 7.6 points. The intervention group demonstrated a reduction of 19.85 points in cardiac anxiety between baseline and 6 months (31.48 to 11.63), which exceeded the estimated threshold, that is, 7.6, and indicates a substantial, clinically meaningful reduction in cardiac anxiety following the HBCR programme.

### Study limitations

The single-centre setting could be considered a limitation of the study. In addition, outcomes were based on self-reported measures, which may introduce bias. Furthermore, the follow-up duration of 6 months may not fully capture the long-term sustainability of behavioural changes or their potential impact on recurrent cardiovascular events.

### Generalisability of findings

Although the randomised controlled trial was conducted at a single site in Lahore, several points may be considered with respect to the generalisability of the study findings. First, the intervention incorporated a culturally sensitive approach, including a patient education booklet in Urdu and active involvement of caregivers, both of which may enhance accessibility and acceptability across different regions. Given that Urdu is the national language and widely understood throughout the country, and that family centred care is a common practice in Pakistani society, the HBCR intervention has strong potential for adaptation and implementation in other provincial settings. These features support the feasibility of scaling the intervention beyond the initial study site. The study findings could potentially apply to similar heterogeneous groups of participants, such as male and female patients with acute coronary syndrome (ACS) aged between 18 years and 65 years who have a diagnosis of STEMI, NSTEMI or angina, and who are treated non-surgically.

While the results of the current RCT can potentially be generalised to similar settings where Urdu is a common language, transfer of full intervention to other settings may not be possible, and further research is needed to adapt interventions for patients and settings where Urdu is not commonly spoken.

### Scalability

The scalability of HBCR in LMICs, such as Pakistan, requires careful consideration of health system capacity, funding mechanisms and sociocultural factors. Although HBCR is promoted as cost-effective and accessible, practical barriers remain. The limited digital infrastructure and variable literacy rates in LMICs can constrain remote monitoring or telehealth components of HBCR programmes. Additionally, there is a need for policies that integrate HBCR into primary healthcare, ensuring sustainable funding and workforce training. Without such structural support, scaling HBCR risks inequitable access, favouring urban or higher socioeconomic groups. Thus, while HBCR presents an innovative solution for cardiac rehabilitation gaps, its expansion demands strategic planning, resource allocation and policy alignment to achieve equitable implementation. However, these contextual factors also highlight the potential value of HBCR in settings where CBCR services are limited or inaccessible. The intervention model used in this study relied on relatively low-cost components, including structured education, behavioural counselling, telephonic follow-up and mobile messaging support, which may be feasible to implement in other resource-constrained healthcare systems. Future multicentre studies conducted across diverse healthcare environments would help further evaluate the scalability and external validity of HBCR programmes.

## Conclusion

The findings of this randomised controlled trial suggest that HBCR may improve heart-related QoL, cardiac health behaviours and cardiac anxiety among patients with coronary artery disease. These findings highlight the potential role of HBCR as an accessible rehabilitation model in settings where participation in CBCR is limited. Further studies with larger sample sizes and longer follow-up periods are needed to evaluate the long-term clinical impact of HBCR interventions.

## Supplementary material

10.1136/bmjopen-2025-114349 online supplemental file 1

## Data Availability

Data are available upon reasonable request.
