# Supplementary figures and images for: Effect of home-based cardiac rehabilitation on quality of life, health behaviours and cardiac anxiety in patients with coronary artery disease: findings from a single-blinded randomised controlled trial
Source: BMJ Open. 2026 Jun 30;16(6):e114349. doi: 10.1136/bmjopen-2025-114349 (PMC13330954; doi:10.1136/bmjopen-2025-114349)

**Histograms**


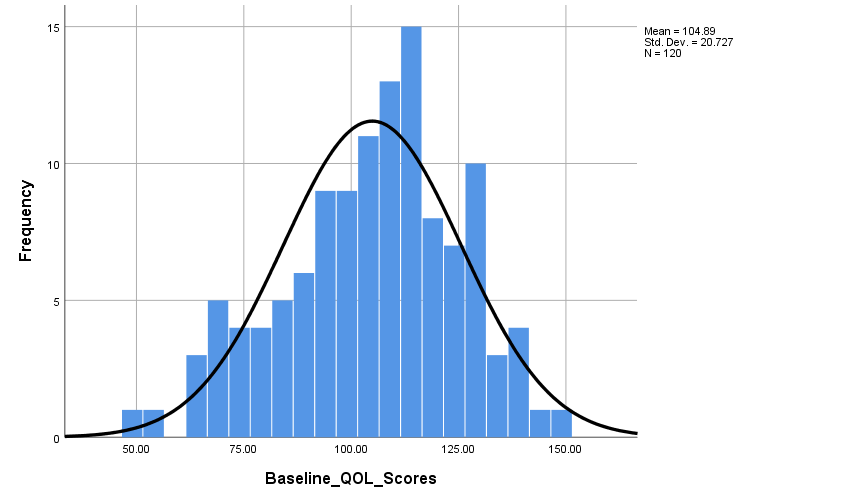

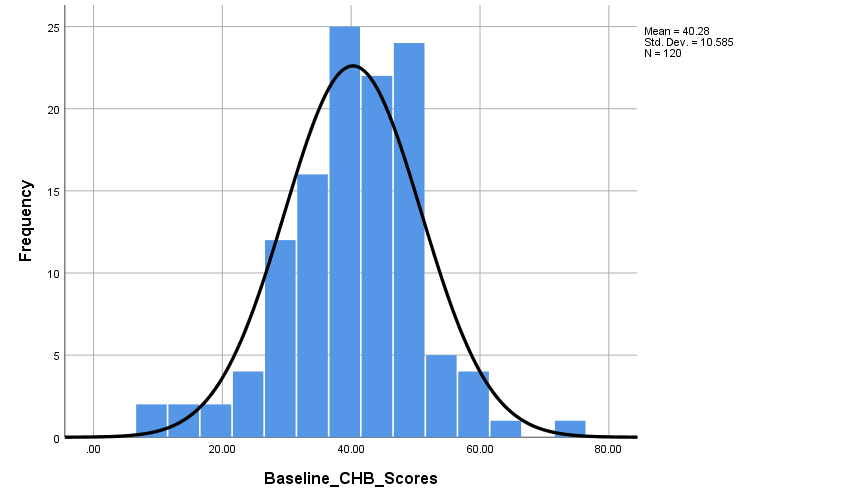

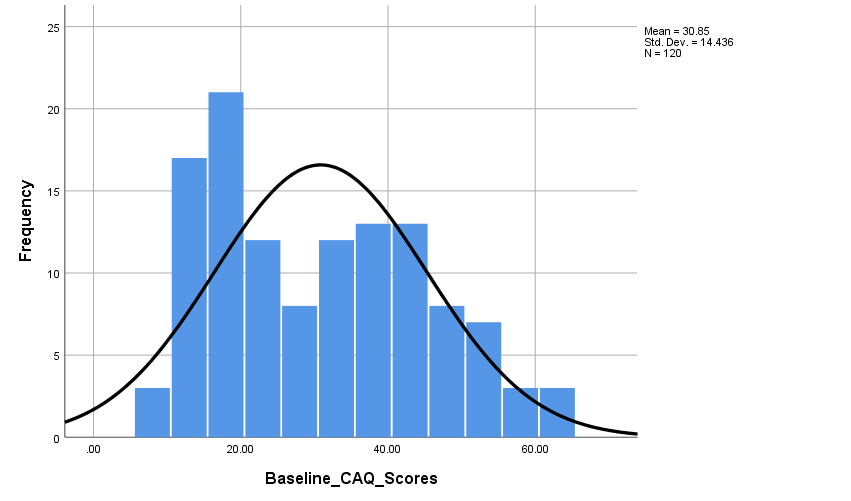

Supplement: online supplemental file 1 [file bmjopen-16-6-s001.docx]
