# Supplementary material for: Effect of home-based cardiac rehabilitation on quality of life, health behaviours and cardiac anxiety in patients with coronary artery disease: findings from a single-blinded randomised controlled trial
Source: BMJ Open. 2026 Jun 30;16(6):e114349. doi: 10.1136/bmjopen-2025-114349 (PMC13330954; doi:10.1136/bmjopen-2025-114349)
Supplement: Supplementary file 2 [file bmjopen-2025-114349.reviewer_comments.pdf]

# PEER REVIEW HISTORY

BMJ Open publishes all reviews undertaken for accepted manuscripts. Reviewers are asked to complete a checklist review form and are provided with free text boxes to elaborate on their assessment. These free text comments are reproduced below.

## ARTICLE DETAILS

### Title (Provisional)

Effect of home-based cardiac rehabilitation on quality of life, health behaviours, and cardiac anxiety in patients with coronary artery disease: findings from a single-blinded randomised controlled trial

### Authors

YAQOOB, Adnan; Ladak, Laila; Khan, Aamir Hameed; Almas, Aysha; Hanif, Asif; Pannu, Furqan Yaqub; Khemani, Faizan; Sahar, Wajeeha; Barolia, Rubina

---

## VERSION 1 - REVIEW

---

| | |
|---|---|
| **Reviewer** | **1** |
| **Name** | **Dack, Kyle** |
| **Affiliation** | **University of Bristol, MRC Integrative Epidemiology Unit** |
| **Date** | **01-Dec-2025** |
| **COI** | **None** |

---

Statistical review:

The study reports that a home based cardiac rehabilitation program improved QoL compared to no further rehabilitation. I do not think there are major sources of bias in the analysis – it would be surprising to not find an improvement given the control group doesn't involve specific planned rehabilitation.

However, there are major omissions from CONSORT guidelines, in the methods and results. Major issues include a lack of distinction between primary/secondary outcomes, a lack of information in the data analysis section, and I am unsure if the reported effect sizes are accurate or how they were calculated. I recommend checking the elaboration document and adding more details to adhere to each section. One potential source of bias is that the analysis hinges on the two outcomes being normally distributed, so I suggest demonstrating this more strongly (e.g. add histograms, or references to prior studies).

Abstract:

1. "Coronary artery diseases possess a major burden of non-communicable diseases" it would be more accurate to rephrase as "..diseases represent a large proportion of non-communicable diseases"

2. CONSORT has specific guidelines for abstracts and subheadings which I recommend applying to ensure clarity, as some key information is missing: https://www.equator-network.org/reporting-guidelines/consort-abstracts/ - Specifically details regarding randomization, blinding, outcome measures, and effect estimates.

Introduction:

3. HBCR should be defined on first use in the introduction. It would be helpful to add more context on what it is, whether there are any existing such interventions, and any evidence perhaps from other contexts, so that readers have a better understanding of the rationale of this intervention and where it came from.

4. CONSORT item 7: Specific objectives and aims are missing, including whether there is a distinction between primary and secondary.

5. As far as I can tell, the primary outcome of this study appears to be QoL (it is presented first, it is what the sample size was based upon). However, this outcome is not mentioned in the introduction, which focuses on the prevention of recurrent cardiac events. Readers will want to know how CR and the intervention are proposed to link to QoL.

Methods:

6. Trial setting – I suggest adding the study dates and the total length of followup, as these are not currently in the methods.

7. Outcomes – please clarify if all three outcomes are intended to be primary, or if some are secondary. The study sample size was based only on QoL which suggests this is the primary outcome.

8. QoL outcome: Please add information on the domain breakdown, as the analysis included comparisons by domain as well as overall.

9. Cardio health behaviours: This section mentions CVI but it is not defined, and I could not follow what was meant by distributing the CVI scale to 5 experts and administering it to 20 patients. Does this mean that this a modified scale which was piloted? If so, I suggest rephrasing to clarify this. Please clarify how the final tool was quantified and aggregated.

10. HARMS: Please add details on how this was intended to be quantified.

11. Sample size: What was the justification for the assumed effect sizes in the intervention and control groups? References are provided but these should be elaborated on in-text. What method was used to calculate the sample sizes? It is important to note that this is powered only for overall QoL, and not the QoL subdomains or cardiac health behaviours. The QoL subdomains seem likely to be underpowered and therefore the interpretation should be cautious, it sounds to me like these are secondary outcomes?

12. My primary statistical concern is that the two outcomes are both summed likert scales. Are there any references showing prior studies performing this? I can see that tests were performed to evaluate normality (please describe this in the data analysis methods section), it may also help to present histograms showing the distribution.

13. Data analysis: This section lacks significant details regarding the comparisons made, and I recommend referring to the CONSORT elaboration document. Broadly, please describe each comparison – including the variables compared, tests used, and assumptions checked.

Results

14. Tables 1 and 2: These are both presenting baseline characteristics and I suggest could be merged.

15. What were the followup results, e.g. attrition at 90 and 180 days?

16. Table 2. Significance testing of baseline characteristics is not suitable because we already know that the differences are due to random chance, which makes the p-value lack meaning: https://link.springer.com/article/10.1186/s12966-015-0162-z

17. “The effect size was large (0.79), demonstrating that 79% of the variation in emotional QoL scores is explained by the HBCR.” Is the effect size not the 180 day outcome score minus the baseline score, compared between groups? This would be (69.06 – 43.23) – (57.81-40.88) which is a 28 point difference. This comment also applies to the effect size reported in later paragraphs and the explained variance, and Table 4.

18. Table 4. Please add the N, statistical tests performed, and replace the 0.00 p-values with a cutoff such as <0.001, as 0.00 is not possible.

19. It is not clear if this analysis was applied on an ITT or complete case basis, and no adverse event results are reported. I recommend rechecking against CONSORT 2025 to ensure all items are fully described.

Discussion

20. The discussion reports several prior studies of HBCR – it would be useful for these to be first introduced in the introduction, as currently that section lacks prior scientific context regarding the intervention.

---

| | |
|---|---|
| **Reviewer** | **2** |
| **Name** | **Cacciapuoti, Fulvio** |
| **Affiliation** | **Antonio Cardarelli Hospital, Cardiology** |
| **Date** | **23-Jan-2026** |
| **COI** | **None** |

---

This is an interesting manuscript examining the effects of a home-based cardiac rehabilitation program on quality of life and cardiac health behaviors. The topic is timely and highly relevant, particularly in settings where access to center-based cardiac rehabilitation is limited. The randomized controlled design and focus on patient-centered outcomes are commendable strengths of the study.

Overall, the manuscript is clearly written and presents findings that may contribute meaningfully to the growing literature on alternative models of cardiac rehabilitation.

However, I would suggest some areas of improvement:

- The manuscript describes the study as “single-blinded,” but it is unclear who was blinded and how blinding was maintained. In addition, the method of allocation concealment is insufficiently described.

Please clarify the randomization procedure, allocation concealment method, and blinding strategy in greater detail.

- While the general components of the HBCR intervention are described, the level of detail is insufficient to allow replication. Information on intervention frequency, duration, intensity, personnel training, and fidelity monitoring is limited.

Consider expanding the intervention description to improve transparency and reproducibility.

- The statistical methods are generally appropriate; however, important details are missing.

Please clarify whether analyses were conducted on an intention-to-treat basis and how missing data were handled.

Reporting and discussing effect sizes and confidence intervals alongside p-values would strengthen interpretation.

Where possible, comment on the clinical relevance of the observed changes (e.g., minimal clinically important differences).

- The discussion appropriately summarizes the findings but would benefit from deeper engagement with issues of generalizability.

Please expand on the implications of the single-center design and contextual factors (e.g., socioeconomic or health system constraints) that may affect scalability and external validity.

---


| | |
|---|---|
| **Reviewer** | **3** |
| **Name** | **Mariani, Javier** |
| **Affiliation** | **GESICA Foundation, Buenos Aires** |
| **Date** | **27-Jan-2026** |
| **COI** | **None** |


---

The manustript reports the results of a RCT assessing the effects of a cardiac rehabilitarion at home program as compared to usual care on QoL and "cardiac behaviors" at 6 months after ACS.

Comments:

Introduction

- There are several statement, like "One of the main factors involved in the failure to prevent recurrent cardiac events after initial treatment is the lack of secondary prevention strategies, such as Cardiac Rehabilitation (CR)", that should be referenced.

- Please, include some background/rational on home-based cardiac rehabilitation as justification for the trial.

- The aim of the study could be the last sentece of the introduction section, it is not necessary to explicit H0 and H1 in a specific section.

Methods

- The Inclusion criteria subsection has the exclusion criteria. Please list all inclusion/exclusion criteria or cite the protocol or apendix with them.

- The subsection "HARMS No physcial harm, such as chest pain and injury, was reported by participants in the intervention group, which could require emergency admission.", could be in the Results section as "adverse events".

- Please, explain what was the rational for the mean difference in QoL used for sample size calculation (is this difference a minimally clinically relevant difference?).

- The subsection "Randomization" actually explain the concealment. Please, explain the random generation, the assignation and the concealment (were the envelopes opaques?).

Results

- The first sentence of results should be in Statistics section.

- A p value <0.05 in KS test actually rule out normality.

- "Therefore, based on the normal distribution of the data, parametric tests such as Repeated Measures ANOVA were used to test differences between groups", consider that normality is just one of several assumptions of RM ANOVA, please revise.

- Second paragraph (describing the sample) should be the first.

- The age is despicted in table 2 and table 3. Please use one table for baseline characteristics.

- Please report the results of the ANOVA model using the p value for the intervention. Reporting 3 p values, one for every time point is misleading. Alternatively, use mixed models.

- The paragraph "Therefore, with 95% confidence, we reject the null hypothesis that states "There is no difference in the QoL and health behaviors of patients with CADs who received usual care with and without HBCR." We conclude that there is a difference in QoL in both groups and that the QoL in the intervention group improved with a more pronounced effect than in the control group, as evidenced by a p-value <0.001." could be supressed from "Results" section.

Discusion

- Please, contextualize your findings to the clinical meaning of the differences in QoL and cardiac behaviors. That is, what these differences imply or could imply in recurrent events.

- There some important limitations not addressed: the sample size, the outcomes (QoL and self-reported behaviors in an open trial -i.e. no cotinine measure for tobacco withdrawal component), and the 6 months follow-up.

- There were no recurrent events during the entire follow-up? There were no adverse events at all (no one re-admission?).

Conclusion

- The statement "The current study suggests that HBCR could significantly improve patient outcomes including QoL and CHB in CAD patients in Pakistan", is not consistent with the results, there were no clinical outcomes assessed, the only reported outcomes were QoL and CHB.

- The sentence "These interventions can be effective in alleviating the long-term risks of heart disease and improving the overall well-being of patients with CAD" is not supported by the study (nor the risks were reduced nor long term assessed).

- The sentences "On the other hand, the provision of an HBCR booklet with routine care also improved patients' QoL and CHB am among the control group" and "he provision of booklets with proper discharge education can also help improve patients' outcomes after MI until the HBCR scale-up in Pakistan" were not proved in the current study. Please revise all conclusions.

---

## VERSION 1 - AUTHOR RESPONSE

| **Reviewer 1:**<br>Kyle Dack, University of Bristol | | | |
|---|---|---|---|
| **10.** | Statistical review:<br>The study reports that a home-based cardiac rehabilitation program improved QoL compared to no further rehabilitation. I do not think there are major sources of bias in the analysis – it would be surprising to not find an improvement given the control group doesn't involve specific planned rehabilitation.<br>However, there are major omissions from CONSORT guidelines, in the methods and results. Major issues include a lack of distinction between primary/secondary outcomes, a lack of information in the data analysis section, and I am unsure if the reported effect sizes are accurate or how they were calculated. I recommend checking the elaboration document and adding | We thank the reviewer for the careful statistical review and helpful suggestions. Following this comment, the manuscript has been thoroughly revised to improve adherence to the CONSORT reporting guidelines.<br>First, the primary outcomes have now been clearly specified in the Methods section. In accordance with the trial protocol and registry record (ACTRN12623000049673p), the primary outcomes were defined as heart-related quality of life, cardiac health behaviours, and cardiac anxiety, assessed at baseline, 3 months, and 6 months, with the 6-month follow-up considered the primary endpoint.<br>Second, the statistical analysis section has been expanded to provide clearer details regarding the analytical approach, including the tests used to | Page # 5, 7C8, table 3<br><br>Supplementary file |

| | | | |
|---|---|---|---|
| | more details to adhere to each section. One potential source of bias is that the analysis hinges on the two outcomes being normally distributed, so I suggest demonstrating this more strongly (e.g. add histograms, or references to prior studies). | assess between-group differences and longitudinal changes over time. Third, to improve transparency of the results, we have added between-group mean differences with corresponding 95% confidence intervals, as recommended by CONSORT guidelines. The effect size estimates have been removed from the results tables to avoid potential ambiguity. Instead, the results are now reported as between-group mean differences with corresponding 95% confidence intervals, in accordance with CONSORT recommendations.. Finally, to address the suggestion regarding normality assumptions, we have clarified that the distribution of the outcome variables was assessed using the Kolmogorov–Smirnov test, and the results indicated that the data were approximately normally distributed. This has now been described more clearly in the revised manuscript. Histograms have been added as supplementary figures | |
| **11.** | Abstract: "Coronary artery diseases possess a major burden of non-communicable diseases." It would be more accurate to rephrase as "..diseases represent a large proportion of non-communicable diseases." | Thank you for this suggestion. Following the editor's request, the abstract has been completely revised to follow the structured format recommended in the *BMJ Open* Instructions for Authors. During this revision, the sentence referenced in the comment was removed from the abstract, and therefore, this | Page # 2 |

| | | | |
|---|---|---|---|
| | | issue has been addressed. However, the sentence in the Introduction has been revised accordingly to improve clarity and accuracy. | |
| **12.** | CONSORT has specific guidelines for abstracts and subheadings, which I recommend applying to ensure clarity, as some key information is missing: https://www.equator-network.org/reporting-guidelines/consort-abstracts/<br>- Specifically details regarding randomization, blinding, outcome measures, and effect estimates. | Thank you for this comment. The abstract has been completely revised to follow the structured abstract format recommended in the *BMJ Open* Instructions for Authors, which is aligned with the CONSORT guidelines for abstracts. The revised abstract now clearly reports the study design (randomised controlled trial), participants, intervention, primary outcome measures, and effect estimates with corresponding 95% confidence intervals, as well as the trial registration number. | Page # 2 |
| **13.** | Introduction:<br>HBCR should be defined on first use in the introduction. It would be helpful to add more context on what it is, whether there are any existing such interventions, and any evidence perhaps from other contexts, so that readers<br>have a better understanding of the rationale of this intervention and where it came from. | Thank you for this suggestion. The introduction has been revised to clearly define home-based cardiac rehabilitation (HBCR) at its first use. | Page # 3 |
| **14.** | CONSORT item 7: Specific objectives and aims are missing, including whether there is a distinction between primary and secondary. | Thank you for highlighting this point. The Introduction has been revised to clearly state the specific objectives and primary outcomes, in accordance with CONSORT reporting recommendations. The primary objectives were to evaluate the effect of the HBCR intervention on heart-related | Page # 3 |

| | | | |
|---|---|---|---|
| | | quality of life, cardiac health behaviours and cardiac anxiety, with the six-month follow-up defined as the primary endpoint. | |
| **15.** | As far as I can tell, the primary outcome of this study appears to be QoL (it is presented first, it is what the sample size was based upon). However, this outcome is not mentioned in the introduction, which focuses on the prevention of recurrent cardiac events. Readers will want to know how CR and the intervention are proposed to link to QoL. | We agree that the link between cardiac rehabilitation and quality of life should be more clearly described in the introduction. We have therefore revised the introduction to explain how cardiac rehabilitation interventions, including home-based programmes, can improve patients' physical functioning, psychological well-being and overall health-related quality of life. | Page # 3 |
| **16.** | Methods:<br>Trial setting – I suggest adding the study dates and the total length of followup, as these are not currently in the methods. | Thank you for pointing this out. The Methods section has been revised to include the study recruitment dates and duration of follow-up. | Page # 4 |
| **17.** | Outcomes – please clarify if all three outcomes are intended to be primary, or if some are secondary. The study sample size was based only on QoL which suggests this is the primary outcome. | Thank you for this observation. In accordance with the published study protocol and trial registration (ACTRN12623000049673p), the study included three primary outcomes: heart-related quality of life, cardiac health behaviours, and cardiac anxiety. | Page # 5 |
| **18.** | QoL outcome: Please add information on the domain breakdown, as the analysis included comparisons by domain as well as overall. | Thank you for this observation. In the initially submitted manuscript, domain-level analyses of the MacNew HRQoL questionnaire (emotional, physical and social domains) were presented. However, to | Page # 8C9 C table 3 |

| | | | |
|---|---|---|---|
| | | simplify the reporting of outcomes and clearly distinguish the primary outcomes, the results were revised to focus on the global QoL score, which represents the overall measure of heart-related quality of life. The domain-level results have therefore been removed from the revised manuscript. | |
| **19.** | Cardio health behaviours: This section mentions CVI but it is not defined, and I could not follow what was meant by distributing the CVI scale to 5 experts and administering it to 20 patients. Does this mean that this a modified scale which was piloted? If so, I suggest rephrasing to clarify this. Please clarify how the final tool was quantified and aggregated. | Thank you for this helpful comment. The description of the Cardiac Health Behaviour Scale (CHB-21) and the Cardiac Anxiety Questionnaire (CAQ-18) has been revised to clarify the process. As validated Urdu versions of these instruments were not available, both scales were translated into Urdu prior to data collection. The translated versions were reviewed by five experts to assess content validity, and the Content Validity Index (CVI) was calculated to evaluate the relevance and clarity of the items. The questionnaires were then pilot tested with 20 patients with coronary artery disease to ensure clarity and feasibility of administration. No substantive modifications were made to the original items. The Methods section has also been revised to clarify how the final scores were calculated and aggregated. | Page # 6 |
| **20.** | HARMS: Please add details on how this was intended to be quantified. | Thank you for this suggestion. The Methods section has been revised to clarify how potential harms were | Page # 6 |

| | | | |
|---|---|---|---|
| | | monitored during the study. This information has now been added to the Methods section. | |
| **21.** | Sample size: What was the justification for the assumed effect sizes in the intervention and control groups? References are provided but these should be elaborated on in-text. What method was used to calculate the sample sizes? It is important to note that this is powered only for overall QoL, and not the QoL subdomains or cardiac health behaviours. The QoL subdomains seem likely to be underpowered, and therefore, the interpretation should be cautious, it sounds to me like these are secondary outcomes? | Thank you for this important observation. Sample size calculations were initially performed for all primary outcomes of the study, including heart-related quality of life, cardiac health behaviours, and cardiac anxiety. Among these outcomes, heart-related quality of life required the largest sample size, and therefore, it was used to determine the final sample size for the trial.<br>The Methods section has been revised to clarify this process. | Page # 6C7 |
| **22.** | My primary statistical concern is that the two outcomes are both summed likert scales. Are there any references showing prior studies performing this? I can see that tests were performed to evaluate normality (please describe this in the data analysis methods section), it may also help to present histograms showing the distribution. | Thank you for this important observation. The outcome measures used in this study, including heart-related quality of life (MacNew HRQoL), cardiac health behaviours (CHB-21), and cardiac anxiety (CAQ-18), generate composite scores by summing multiple Likert-type items. When Likert-type items are aggregated into composite scale scores, it is common practice in health research to treat the resulting variables as approximately continuous and analyse them using parametric statistical methods (cited in text). To address the suggestion, the Statistical Analysis section has been revised to describe how the distribution of outcome | Page # 7 |

| | | | |
|---|---|---|---|
| | | variables was assessed using the Kolmogorov–Smirnov test and visual inspection of histograms. The distributions of the outcome variables were approximately normal, supporting the use of parametric statistical tests. Histograms have been added as supplementary figures | |
| **23.** | Data analysis: This section lacks significant details regarding the comparisons made, and I recommend referring to the CONSORT elaboration document. Broadly, please describe each comparison – including the variables compared, tests used, and assumptions checked. | Thank you for this helpful suggestion. The Statistical Analysis section has been substantially revised to provide clearer details regarding the analytical approach used in the study. The revised section now describes the statistical software used, the descriptive statistics reported, the tests used to compare baseline characteristics between groups, and the analytical approach used to evaluate changes in outcomes over time. We have also clarified how the assumptions of the statistical tests were assessed, including evaluation of the distribution of outcome variables using the Kolmogorov–Smirnov test and visual inspection of histograms. These revisions were made to improve clarity and to ensure closer alignment with CONSORT reporting recommendations. | Page # 7 |
| **24.** | Results<br>Tables 1 and 2: These are both presenting baseline characteristics and I suggest could be merged. | Both tables have been merged in the revised manuscript. | Table 2 |

| | | | |
|---|---|---|---|
| **25.** | What were the followup results, e.g. attrition at 90 and 180 days? | Details have been added in Results section. | Page # 8 |
| **26.** | Table 2. Significance testing of baseline characteristics is not suitable because we already know that the differences are due to random chance, which makes the p-value lack meaning: https://link.springer.com/article/10.1186/s12966-015-0162-z | Thank you for this helpful observation. In line with CONSORT recommendations, statistical significance testing of baseline characteristics is not necessary in randomized controlled trials because any differences between groups are expected to arise from random allocation. The p-values previously presented in the baseline table have therefore been removed, and the table now presents only descriptive statistics for participant characteristics. | Page # 8C9 Table 2 |
| **27.** | "The effect size was large (0.79), demonstrating that 79% of the variation in emotional QoL scores is explained by the HBCR." Is the effect size not the 180 day outcome score minus the baseline score, compared between groups? This would be (69.06 – 43.23) – (57.81-40.88) which is a 28 point difference. This comment also applies to the effect size reported in later paragraphs and the explained variance, and Table 4. | Thank you for this observation. In the initial submission, effect size estimates and interpretations of explained variance were reported in the results section. However, during the revision, these effect size measures were removed to simplify reporting and avoid potential misinterpretation. The revised manuscript now reports intervention effects as between-group mean differences with corresponding 95% confidence intervals, which is consistent with CONSORT reporting recommendations. | Page # 9 Table 3 |

| | | | |
|---|---|---|---|
| **28.** | Table 4. Please add the N, statistical tests performed, and replace the 0.00 p-values with a cutoff such as <0.001, as 0.00 is not possible. | Thank you for this helpful suggestion. Table has been revised to improve clarity and reporting. The sample size (N) has now been added to the table, the statistical test used has been specified, and p-values previously reported as 0.00 have been corrected to <0.001, as recommended. | Table 3 |
| **29.** | It is not clear if this analysis was applied on an ITT or complete case basis, and no adverse event results are reported. I recommend rechecking against CONSORT 2025 to ensure all items are fully described. | Description added in the revised manuscript | Page # 8 |
| **30.** | Discussion<br>The discussion reports several prior studies of HBCR – it would be useful for these to be first introduced in the introduction, as currently that section lacks prior scientific context regarding the intervention. | The introduction section has been revised. | Page # 3 |
| **Reviewer: 2**<br>Dr. Fulvio Cacciapuoti, Antonio Cardarelli Hospital | | | |
| **31.** | This is an interesting manuscript examining the effects of a home-based cardiac rehabilitation program on quality of life and cardiac health behaviors. The topic is timely and highly relevant, particularly in settings where access to center-based cardiac rehabilitation is limited.<br>The randomized controlled design and focus on patient-centered outcomes are commendable strengths of the study. | We sincerely thank the reviewer for the positive and encouraging feedback regarding the relevance of the topic, the randomized controlled design, and the focus on patient-centered outcomes. We appreciate the reviewer's recognition of the potential contribution of this study to the literature on alternative models of cardiac rehabilitation. We have carefully considered the | **-** |

| | | | |
|---|---|---|---|
| | | reviewer's suggestions and | |
| | Overall, the manuscript is clearly written and presents findings that may contribute meaningfully to the growing literature on alternative models of cardiac rehabilitation. However, I would suggest some areas of improvement: | have revised the manuscript accordingly, as detailed below. | |
| **32.** | The manuscript describes the study as "single-blinded," but it is unclear who was blinded and how blinding was maintained. In addition, the method of allocation concealment is insufficiently described.<br>Please clarify the randomization procedure, allocation concealment method, and blinding strategy in greater detail. | Thank you for this comment. The description of the randomization, allocation concealment, and blinding procedures has been expanded in the Methods section to improve clarity. | Page # 7 |
| **33.** | While the general components of the HBCR intervention are described, the level of detail is insufficient to allow replication. Information on intervention frequency, duration, intensity, personnel training, and fidelity monitoring is limited.Consider expanding the intervention description to improve transparency and reproducibility. | To further improve clarity and reproducibility, we have revised the intervention description to more explicitly highlight the roles of the personnel involved in delivering the intervention and the procedures used to monitor intervention fidelity during the follow-up period. | Page # 5 |
| **34.** | The statistical methods are generally appropriate; however, important details are missing. Please clarify whether analyses were | The Statistical Analysis section has been revised to clarify the analytical approach used in the study.<br>Specifically, we have added information describing how missing data were handled using the last | Page # 8 |

| | | | |
|---|---|---|---|
| | conducted on an intention-to-treat basis and how missing data were handled. | observation carried forward (LOCF) method,<br>ensuring that all randomized participants were included in the analysis. | |
| **35.** | Reporting and discussing effect sizes and confidence intervals alongside p-values would strengthen interpretation. | Mean differences and Confidence intervals, along with p-values, have been added. | Page # 9<br>Table 3 |
| **36.** | Where possible, comment on the clinical relevance of the observed changes (e.g., minimal clinically important differences). | Thank you for this valuable suggestion. We have expanded the Discussion section to comment on the clinical relevance of the observed changes. | Page # 10C11 |
| **37.** | The discussion appropriately summarizes the findings but would benefit from deeper engagement with issues of generalizability. | We have expanded the Discussion section to further address the generalizability of the study findings. | Page #11 |
| **38.** | Please expand on the implications of the single-center design and contextual factors (e.g.,<br>socioeconomic or health system constraints) that may affect scalability and external validity. | Thank you for this comment. Details have been added in the discussion section | Page #8, 11, 12 |
| **Reviewer: 3**<br>Dr. Javier Mariani, GEDIC | | | |
| **39.** | The manuscript reports the results of a RCT assessing the effects of a cardiac rehabilitation at home program as compared to usual care on QoL and "cardiac behaviors" at 6 months after ACS. | Thank you, We appreciate the opportunity to revise and improve the manuscript and have addressed the reviewer's comments and suggestions as detailed below. | **-** |

| | | | |
|---|---|---|---|
| **40.** | There are several statement, like "One of the main factors involved in the failure to prevent recurrent cardiac events after initial treatment is the lack of secondary prevention strategies, such as Cardiac Rehabilitation (CR)", that should be referenced. | The introduction section has been revised and appropriate references have been included. | Page #3 |
| **41.** | Please, include some background/rational on home-based cardiac rehabilitation as justification for the trial. | The introduction section has been revised in line with the comment. | Page #3 |
| **42.** | The aim of the study could be the last sentece of the introduction section, it is not necessary to explicit H0 and H1 in a specific section. | The aim has been mentioned at the end of the introduction, hypothesis section has been removed. | Page #3 |
| **43.** | Methods<br>The Inclusion criteria subsection has the exclusion criteria. Please list all inclusion/exclusion criteria or cite the protocol or appendix with them. | Eligibility criteria have been added as suggested | Page #4 |
| **44.** | The subsection "HARMS No physcial harm, such as chest pain and injury, was reported by participants in the intervention group, which could require emergency admission.", could be in the Results section as "adverse events". | The description of harm monitoring has been retained in the Methods section, while the statement regarding the absence of adverse events has been moved to the Results section under a new subsection titled *Adverse events*, in line with CONSORT reporting recommendations. | Page #9 |
| **45.** | Please, explain what was the rational for the mean difference in QoL used for sample size | The expected difference used for the sample size calculation was informed by a previously published | - |

| | | | |
|---|---|---|---|
| | calculation (is this difference a minimally clinically relevant difference?). | cardiac rehabilitation study (Wang et al., 2012), in which the score improved from 50 at baseline to 80.8 at six months, corresponding to an improvement of 30.8 points. Considering that the minimal clinically important difference, the improvement observed in the reference study represents a clinically meaningful change. These data were therefore used to estimate the expected treatment effect for the sample size calculation. | |
| **46.** | The subsection "Randomization" actually explain the concealment. Please, explain the random generation, the assignation and the concealment (were the envelopes opaques?). | The details have been added. | Page #7 |
| **47.** | Results<br>- The first sentence of results should be in Statistics section. | The result section has been revised | Page #8-9 |
| **48.** | A p value <0.05 in KS test actually rule out normality. "Therefore, based on the normal distribution of the data, parametric tests such as Repeated Measures ANOVA were used to test differences between groups", consider that normality is just one of several assumptions of RM ANOVA, please revise. | Thank you for this helpful comment. We have revised the Statistical Analysis section to clarify that the use of repeated-measures ANOVA was based on the evaluation of several statistical assumptions, not solely the normality of the data. | Page #7 |
| **49.** | Second paragraph (describing the sample) should be the first. | The result section has been revised | Page # 8C9 |

| | | | |
|---|---|---|---|
| **50.** | The age is despicted in table 2 and table 3.<br>Please use one table for baseline characteristics. | Tables have been merged | Table 2 |
| **51.** | Please report the results of the ANOVA model<br>using the p value for the intervention. Reporting 3 p values, one for every time point is misleading. Alternatively, use mixed models. | Thank you for this helpful suggestion. We have<br>revised the Results section to report the results from the repeated-measures ANOVA model rather than separate p-values at each time point. | Page # 7C8 C<br>Table 3 |
| **52.** | The paragraph "Therefore, with 95% confidence, we reject the null hypothesis that states "There is no difference in the QoL and health behaviors of patients with CADs who received usual care with and without HBCR." We conclude that there is a difference in QoL in both groups and that the QoL in the intervention group improved with a more pronounced effect than in the control group, as evidenced by a p-value <0.001." could be supressed from "Results" section. | Suggested paragraph has been removed from results section. | Page # 9 |
| **53.** | Discusion<br>Please, contextualize your findings to the clinical meaning of the differences in QoL and cardiac behaviors. That is, what these differences imply or could imply in recurrent events. | MCIDs of each outcome have been reported in the discussion. | Page # 10C11 |
| **54.** | There some important limitations not addressed: the sample size, the outcomes (QoL and self-reported behaviors in an open trial -i.e. no cotinine measure for tobacco withdrawal component), and the 6 months follow-up. | The limitations have been added under the section of Strengths and Limitations. | Page # 2 |

| | | | |
|---|---|---|---|
| **55.** | There were no recurrent events during the entire follow-up? There were no adverse events at all (no one re-admission?). | At 6 months, intervention group two had emergency visits due to non-cardiac reasons, as compared to ten in the control group. Reduction in emergency visits in the intervention group in the current study is attributed to improved patient interaction with the researcher and HCPs, telephonically addressed health concerns with the help of a cardiologist, physical health, weight loss, and better control of cardiac risk factors, all of which are optimized through HBCR with enhanced participation over six months. The details have been added in results section under adverse events heading. | Page # 9 |
| **56.** | Conclusion<br>The statement "The current study suggests that HBCR could significantly improve patient outcomes including QoL and CHB in CAD patients in Pakistan", is not consistent with the results, there were no clinical outcomes assessed, the only reported outcomes were QoL and CHB. The sentence "These interventions can be effective in alleviating the long-term risks of heart disease and improving the overall well-being of patients with CAD" is not supported by the study (nor the risks were reduced nor long term assessed). The sentences "On the other hand, the provision of an HBCR booklet with routine care also improved patients' QoL and CHB am among the control group" and | The conclusion section has been revised. | Page # 12 |

| | | | |
|---|---|---|---|
| | "he provision of booklets with proper discharge education can also help improve patients' outcomes after MI until the HBCR scale-up in Pakistan" were not proved in the current study. Please revise all conclusions. | | |

## VERSION 2 - REVIEW

| | |
|---|---|
| **Reviewer** | **1** |
| **Name** | **Dack, Kyle** |
| **Affiliation** | **University of Bristol, MRC Integrative Epidemiology Unit** |
| **Date** | **09-Apr-2026** |
| **COI** | |

The authors made substantial revisions to apply the CONSORT guidelines, which fully addressed all my prior queries or comments. Concerns I had regarding the outcomes and sample size are now also resolved, and I have no further queries.

Minor formatting notes:

In the abstract and main results section, the point estimates are reported to 3 decimal places, but the 95% CI to 2 decimal places - it would look better if both were consistent.

Table 2. Readers may not understand that f refers to frequency, and it would be beneficial to add a footnote defining this. Alternatively I think it would be just as clear without the "f=" part.

| | |
|---|---|
| **Reviewer** | **3** |
| **Name** | **Mariani, Javier** |
| **Affiliation** | **GESICA Foundation, Buenos Aires** |
| **Date** | **07-May-2026** |
| **COI** | |

The authors addressed all comments.

## VERSION 2 - AUTHOR RESPONSE

| | | | |
|---|---|---|---|
| 1 | Reviewer: 1<br>Dr. Kyle Dack, University of Bristol Comments to the Author:<br>The authors made substantial revisions to apply the CONSORT guidelines, which fully addressed all my prior queries or comments. Concerns I had regarding the outcomes and sample size are now also resolved, and I have no further queries. | We sincerely thank the reviewer for the positive feedback and critical evaluation of our manuscript. | - |
| 2 | Minor formatting notes:<br>In the abstract and main results section, the point estimates are reported to 3 decimal places, but the 95% CI to 2 decimal places - it would look better if both were consistent. [NOTE FROM THE EDITORS: We agree with this and suggest reporting all data to two decimal places throughout, including the abstract, main text, and tables.<br>Please ensure all data are reported consistently between these different locations] | Thank you for this helpful suggestion. We carefully reviewed the abstract, main text, tables to ensure consistency throughout the manuscript. All point estimates, standard deviations, confidence intervals, and related statistical values are now presented consistently to two decimal places in accordance with the journal's recommendation. | 2, 9-10, Tables 1&2 |

| | | | |
|---|---|---|---|
| 3 | Table 2. Readers may not understand that f refers to frequency, and it would be beneficial to add a footnote defining this. Alternatively I think it would be just as clear<br>without the "f=" part. | Thank you for this helpful suggestion. We have revised Table 2 by removing the "f=" notation to improve readability and clarity for readers. | Tables 2 |
| 4 | Reviewer: 3<br>Dr. Javier Mariani, GESICA Foundation, Buenos Aires Comments to the Authors:<br>The authors addressed all comments. | We sincerely thank the reviewer for the positive feedback and thoughtful evaluation of our manuscript. | - |
